# HLA Polymorphisms and Clinical Manifestations in IgA Vasculitis

**DOI:** 10.3390/ijms25020882

**Published:** 2024-01-10

**Authors:** Martina Held, Katarina Stingl Jankovic, Mario Sestan, Matej Sapina, Nastasia Kifer, Sasa Srsen, Marijan Frkovic, Alenka Gagro, Zorana Grubic, Marija Jelusic

**Affiliations:** 1 Department of Pediatrics, University Hospital Centre Zagreb, University of Zagreb School of Medicine, 10000 Zagreb, Croatia; martina.held@mef.hr (M.H.);; 2 Tissue Typing Centre, Clinical Department for Transfusion Medicine and Transplantation Biology, University Hospital Centre Zagreb, 10000 Zagreb, Croatiazorana.grubic@kbc-zagreb.hr (Z.G.); 3 Department of Pediatrics, University Hospital Centre Osijek, Josip Juraj Strossmayer University of Osijek, Medical Faculty Osijek, 31000 Osijek, Croatia; sapina.matej@yahoo.com; 4 Department of Pediatrics, University of Split School of Medicine, University Hospital Centre Split, 21000 Split, Croatia; ssrsen@kbsplit.hr; 5 Children’s Hospital Zagreb, Medical Faculty Osijek, Josip Juraj Strossmayer University of Osijek, 10000 Zagreb, Croatia; alenka.gagro@gmail.com

**Keywords:** IgA vasculitis, human leukocyte antigen polymorphisms, allele frequencies, high-resolution typing, HLA-DRB1, next generation sequencing, Croatians

## Abstract

Studies concerning the genetic background of IgA vasculitis (IgAV), a small-vessel vasculitis occurring predominantly in childhood, have confirmed that the HLA-DRB1 gene showed a strong association with disease susceptibility. The objective was to investigate human leukocyte antigen (HLA) polymorphisms among Croatian patients with IgAV and their influence on disease susceptibility and clinical heterogeneity. Thus, 130 children with IgAV and 202 unrelated healthy individuals were enrolled in the study. Genomic DNA was extracted from whole peripheral blood, and HLA-A, -B, -DRB1 and -DQB1 gene polymorphism analysis was performed. HLA-A*03 (21.4% vs. 12.38%, *p* = 0.0092), HLA-B*37 (2.9% vs. 0.2%, *p* = 0.0054) and HLA-DRB1*12 (3.1% vs. 0.7%, *p* = 0.0216) alleles were significantly more frequent in IgAV patients than in controls. High-resolution typing revealed significantly higher frequency of HLA-DRB1*10:01 and -DRB1*11:03 among IgAV patients with gastrointestinal manifestations of the disease in comparison to controls (*p* = 0.0021 and *p* = 0.0301, respectively), while HLA-DRB1*14:01P occurred significantly more often in the group of patients who developed nephritis during the course of the disease (17.5% vs. 4.5%, *p* = 0.0006). Our results demonstrated that there is an association of HLA-A*03, HLA-B*37 and HLA-DRB1*12 alleles with susceptibility to IgAV in the examined Croatian pediatric population. Studies which aim to determine the HLA profile may contribute to the elucidation of the genetic background of autoimmune diseases, including IgAV.

## 1. Introduction

IgA vasculitis (IgAV), formerly known as Henoch-Schönlein purpura, is the most common systemic childhood vasculitis, with an estimated worldwide annual incidence of 3–55.9 cases per 100,000 children [1,2,3]. Using precise geostatistical methods, the average annual incidence of IgAV in the Republic of Croatia is estimated to be 6.79 per 100,000 children, which is similar to other European countries [2]. The main feature of IgAV is a palpable purpura or petechiae, predominantly affecting lower extremities accompanied by arthritis and/or arthralgias, abdominal pain and IgA vasculitis nephritis (IgAVN) [4]. IgAV is usually a self-limiting disease that lasts up to four weeks, with a prognosis that almost exclusively depends on the developed acute or chronic complications. Severe gastrointestinal and systemic manifestations refer to acute complications of the disease, while renal involvement is the most significant chronic complication and the main cause of morbidity and mortality among affected children [2,5,6]. According to recently published studies, children with severe cutaneous manifestations such as ulcerations and necrosis and purpuric rash that persists for more than a month, older age and with severe gastrointestinal symptoms (severe abdominal pain, intussusception, hematochezia and/or massive gastrointestinal bleeding) of IgAV are a particularly high-risk group for developing IgAVN [5,7].

A multifactorial ethiopathogenesis of IgAV has been proposed, in which various genetic and environmental factors trigger the disease. When comparing genetic variants between healthy individuals and IgAV patients in susceptibility to the disease, significant differences are found in human leukocyte antigen (HLA) genes. These genes encode molecules, which have a crucial role in presenting antigenic peptides to T cells and, therefore, facilitating the immune system to differentiate between self and non-self [8]. The HLA complex has been extensively studied for association with numerous diseases, and in the case of IgAV, previous studies have mostly pointed out the association with HLA class II genes, in particular the HLA-DRB1 locus [9,10,11,12,13].

In three genome-wide association studies (GWAS) so far, the first of them revealed the impact of the polymorphisms in the HLA-DQA1 and HLA-DQB1 intergenic zone and at the HLA-DRB1 locus (HLA-DRB1*11 and DRB1*13), and, more importantly, the second determined that the haplotype HLA-DRB1*01:01~DQB1*05:01~DQA1*01:01 was only associated with susceptibility to IgAV but not with other autoimmune diseases, highlighting that IgAV is a prototype of HLA class II disease. The third confirmed the strong association of HLA-DRB1 with susceptibility to IgAV [14,15,16]. The possible influence of HLA class I genes has also been investigated, reporting mixed results, probably due to the smaller sample size of the investigated cohort [15,17,18,19,20]. Apart from susceptibility to the disease itself, HLA class I gene polymorphisms may also affect clinical heterogeneity and disease severity, confirming the complexity of the genetic contribution in understanding the pathogenesis, course and outcome of IgAV.

For this purpose, in this study, we examined whether HLA-A, -B, -DRB1 and -DQB1 polymorphisms have an influence on the susceptibility and different phenotypes within the clinical course in a population of Croatian children with IgAV.

## 2. Results

The present study included 130 patients with IgAV and 202 control subjects for whom the HLA-A, -B, -DRB1 and -DQB1 polymorphism was analyzed. Information about the patients’ gender, age at disease onset and clinical features is shown in Table 1. Out of 130 children with IgAV, 71 were girls and 59 were boys, with a median age of 6.3 (4.4–8.1) years at the time of diagnosis. All patients had purpuric rash, 108 patients (83.1%) had joint involvement, 50 patients (38.5%) had gastrointestinal (GI) tract involvement, while 39 (30.0%) patients developed IgA vasculitis nephritis (IgAVN) during the course of the disease.

The results of the HLA-A, -B, -DRB1 and -DQB1 loci typing at the gene level (low-resolution level) in the entire patient group (N = 130) as well as among controls (N = 202) are presented in Table 2.

The HLA-A and -B typing results were available for 70 patients, HLA-DQB1 gene frequencies were determined for 91 patients, while the HLA-DRB1 locus was analyzed for all 130 IgAV patients.

Among sixteen different genes detected at the HLA-A locus, the most frequent one was HLA-A*02, both in the patient group and among controls (24.3% and 31.2%, respectively). The only significant difference in the frequency distribution of HLA-A genes between IgAV patients and healthy controls was observed for the HLA-A*03 gene, which was significantly more present among patients (21.4% vs. 12.38%, *p* = 0.0092, 95% CI—2.1% to 17.1%).

The HLA-B locus is characterized with a higher polymorphism level in comparison to other HLA class I loci, and this was proven by our results, with 28 different HLA-B genes detected in total in both study cohorts (25 different HLA-B genes among patients and 27 among controls). As opposed to the HLA-A locus, the gene frequencies at the HLA-B locus are more evenly distributed, with HLA-B*35 being the most frequently detected gene among IgAV patients, followed by HLA-B*44 and -B*51 genes (15.0%, 11.4% and 10.7%, respectively). In contrast, HLA-B*51, -B35 and -B*07 were the most frequent HLA-B genes in the control group (12.1%, 11.6% and 9.4%, respectively). This difference, however, was not significant. The only notable difference was detected for the presence of a less commonly found HLA-B gene in the Croatian population, the HLA-B*37 gene, which was present in 2.9% of IgAV patients but only among 0.2% of control subjects (*p* = 0.0054, 95% CI—0.51% to 6.9%).

The distribution of gene frequencies for the thirteen detected HLA-DRB1 genes also differed between IgAV patients and control subjects. The most frequent gene in the patient groups was the HLA-DRB1*11 gene (15.4%), as opposed to controls who carried the HLA-DRB1*13 gene in the majority of cases (16.3%). The only significant difference was calculated for the presence of the HLA-DRB1*12 gene, which occurred with a frequency of 3.1% in the patient group but only in 0.7% of controls (*p* = 0.0216, 95% CI—0.3% to 5.3%).

Finally, the HLA-DQB1 typing results analysis revealed seven different genes, with HLA-DQB1*05 being the most frequent HLA-DQB1 gene in both patient and control groups (31.9% and 25.7%, respectively). No significant difference in gene frequency for any of the observed HLA-DQB1 genes was found.

In order to determine the possible influence of the HLA-DRB1 polymorphism on different clinical manifestations of IgAV, the patient group was also divided into four subgroups: patients with GI involvement (IgAV and GI, N = 31), patients with nephritis (IgAVN, N = 20), patients with both GI involvement and nephritis (IgAVN and GI, N = 19) and patients without GI involvement and nephritis (IgAV, N = 60). The comparison of HLA-DRB1 allele frequencies found in the entire patient group and subgroups to those observed among controls, as well as the comparison of HLA-DRB1 allele frequencies distribution in different patient subgroups, are presented in Table 3 and Table 4. Only those HLA-DRB1 alleles for which a significant difference in frequency was observed in one of the comparisons are listed.

The analysis of high-resolution HLA typing results in addition to the division of patients into subgroups according to the presence of GI involvement and nephritis revealed several additional HLA-DRB1 alleles demonstrating association with IgAV. More precisely, HLA-DRB1*10:01 and -DRB1*11:03 were significantly more present among IgAV and GI patients in comparison to controls (*p* = 0.0021 and *p* = 0.0301, respectively), while HLA-DRB1*14:01P occurred significantly more often in the IgAVN group of patients than among controls (17.5% vs. 4.5%, *p* = 0.0006; 95% CI—3.9% to 27.6%). The increase in HLA-DRB1*10:01 allele frequency among IgAV and GI patients and -DRB1*14:01P allele frequency among IgAVN patients were also significant in comparison to those observed among patients without GI involvement and nephritis (*p* = 0.0050 and *p* = 0.0242, respectively). A comparison of HLA-DRB1 allele frequencies among IgAV and GI and IgAV patients revealed a significant increase in HLA-DRB1*04:02 allele frequency among patients with GI involvement (*p* = 0.0485). Finally, the alleles HLA-DRB1*04:04 and -DRB1*11:01 were detected significantly more often among patients with both GI involvement and nephritis in comparison to patients without those two clinical manifestations of the disease (*p* = 0.0117 and *p* = 0.0480, respectively). The results of multivariate logistic regression showed that HLA- DRB1*10 was significantly associated with GI symptoms and IgAV (OR = 6.67; 95% CI 1.62–27.49; *p* = 0.009), HLA-DRB1*14, with both IgAVN and IgAV (OR = 5.09; 95% CI 1.95–13.28; *p* = 0.001), and HLA-DRB1*12 with overall IgAV (OR = 5.84; 95% CI 1.37–24.88; *p* = 0.017) compared to controls.

## 3. Discussion

Although the pathogenesis of IgAV remains unclear, it is considered to be an immune complex-mediated disease, in which many genetic, immunological and environmental factors intertwine [21]. It seems that genetic predisposition is almost unquestionable in IgAV development since the disease shows a higher incidence among first-degree relatives of affected patients and sometimes familial aggregation [22,23,24]. However, we did not observe such an association here or for familial clustering, although a familial history of autoimmune rheumatic diseases was noticed among some patients.

We performed the present study to investigate the distribution of HLA class I and class II genes among Croatian children with IgAV and to examine whether these genes have an effect on the susceptibility, severity and clinical heterogeneity of IgAV in our population.

The results of HLA class I gene analysis in our cohort of patients revealed only two genes demonstrating a positive association with the disease, HLA-A*03 and HLA-B*37. The HLA-B*37 has not been identified as a risk factor for IgAV in any previous investigations, while for the HLA-A*03 gene, a positive association with IgAV patients with joint involvement was reported by Peru et al. [17]. This study also implicated HLA-B44 as a risk factor, but, although this gene occurred with an increased frequency among IgAV patients, in the present study, in comparison to controls, this difference did not reach statistical significance. The same observation is valid for the HLA-B*35 gene, which was found to be associated with IgAV among Turkish children [17] as well as among patients from Northwest Spain with renal implications [18]. This frequency of the HLA-B*35 gene was also increased in our IgAV patient group but without statistical significance. The disparity of our results regarding the HLA-B*35 gene could be explained by the small sample size, both in the present study since HLA class I typing was available for 90 IgAV patients but also in the abovementioned studies, where the sample size was 110 and 48 patients, respectively. Similarly, the HLA-B*40 (serological equivalent HLA-B60), described as a protective gene in a Mongolian study [25], was detected with a decreased frequency in our IgAV patient group but insufficiently for a significant association. It is also important to mention population differences between Mongolian and our population regarding the HLA-B frequencies. Previous studies also reported an increased susceptibility to IgAV among HLA-B*41:02-positive Spanish patients, irrespective of HLA-DRB1 status [19] and for HLA-A2- and HLA-A11-positive patients from Turkey [17], but our results did not confirm these findings. Finally, there were also reports, which observed no association of HLA class I antigens and IgAV [20].

As previously reported in GWAS studies, HLA class II genes have been implicated in susceptibility to IgAV, with special emphasis on HLA-DRB1 alleles [14,15,16]. Therefore, IgAV shares some features with giant cell arteritis (GCA) and anti-neutrophil cytoplasmic antibody (ANCA)-associated vasculitis (AAV), as these entities have also shown association with HLA class II genes [26,27,28]. In addition to GWAS studies, several studies evaluated the potential association between the HLA-DRB1 genes and IgAV in different ethnic groups. In the present study, a significant positive association with IgAV was detected for the HLA-DRB1*12 gene. This gene showed a possible susceptibility to autoimmune and rheumatic diseases, such as seronegative rheumatoid arthritis [29], undifferentiated spondyloarthritis [30], dermatomyositis and polymyositis [31,32], granulomatosis with polyangiitis [33] and sarcoidosis [34,35,36], with studies predominantly focused on patients from Asian populations. The association between IgAV and the HLA-DRB1*12 gene was previously reported in a large Chinese GWAS study where a significant independent signal of the HLA-DRB1*12:02 allele was demonstrated [16].

The division of our patient group into four subgroups regarding the presence/absence of GI or renal involvement revealed that this association is limited for patients without either GI involvement or nephritis. The difference in the strength of association shown for the HLA-DRB1*12 gene and HLA-DRB1*12:01 allele lies in the fact that the allelic variants of this gene in the Croatian population are not numerous, and HLA-DRB1*12:01 is found almost exclusively. However, there was an individual carrying the HLA-DRB1*12:02 allele in our patient group, which influenced the statistical calculation. The HLA-DRB1*12 gene has not been reported as a susceptibility factor in any of the previous studies, which, thus far, have described an increased susceptibility to IgAV for HLA-DRB1*01-positive Spanish, Italian and Iranian patients (Italian, Turkish and Iranian HLA-DRB1*11 allele carriers and Turkish patients positive for the HLA-DRB1*14 gene) [9,10,11,12,13]. On the other hand, HLA-DRB1*07 was negatively associated with the disease in the Spanish and Italian cohorts [9,10], while a decreased frequency of HLA-DRB1*10 and HLA-DRB1*03 genes was detected in the Turkish cohorts [13], so these genes seem to have a protective role in these ethnic groups. Both HLA-DRB1*03:01 and -DRB1*07:01 alleles occurred with a decreased frequency in our patient group when compared to the controls, but this decrease did not result in a significant *p* value. The decrease in the HLA-DRB1*03:01 allele’s frequency in our IgAV patient group is an interesting finding in general since this HLA-DRB1 allele has been identified as a risk factor in numerous autoimmune diseases thus far [37,38,39].

Opposingly, the HLA-DRB1*10 gene demonstrated a positive association with IgAV patients with GI involvement in our research. However, this finding has to be proven on a larger patient cohort and compared to data from other populations in order to determine whether this association is characteristic for Croatian patients with IgAV. HLA-DRB1 typing at high resolution revealed one more allele demonstrating a positive association with this clinical manifestation of IgAV, the HLA-DRB1*11:03 allele. Previous studies did not reveal association with the clinical features of IgAV regarding gastrointestinal or renal involvement, with any specific HLA-DRB1 gene [9,10,11,12,13], except for HLA-DRB1*11 with joint manifestations in Turkish children [13]. The same study also observed an increased frequency of the HLA-DRB1*13 gene in children with nephrotic proteinuria [13]. The exception among the conducted studies so far is an Indian one that did not reveal any association of HLA-DRB1 genes with susceptibility to IgAV, nor with clinical features of the disease [40]. Our results regarding the IgAV with renal manifestation indicated a positive association with HLA-DRB1*14:01P (HLA-DRB1*14:01/*14:54), which was implicated as a susceptibility marker in a study by Soylemezoglu et al. [13].

The analysis of our results regarding the HLA class II genes did not reveal any association with HLA-DQB1 genes, as opposed to several previous investigations, which reported the association of HLA-DQB1*05:01, -DQB1*03:01 and -DQB1*02:01 alleles with IgAV [9,15]. The association with HLA-DQB1 alleles, as the authors themselves have stated, might be a result of the linkage disequilibrium (LD) with HLA-DRB1 alleles, which show a primary association in the majority of the, thus far, investigated autoimmune diseases. The HLA-DQB1*05:01 and HLA*DQB1*03:01 are in LD with HLA-DRB1*01 and HLA-DRB1*11 genes, respectively, while HLA-DQB1*02 can be found in combination with HLA-DRB1*03 and -DRB1*07 genes. The positive association of HLA-DQB1*05:01 and HLA*DQB1*03:01 alleles, and negative association of the HLA-DQB1*02 gene (DQB1*02:01 and DQB1*02:02 alleles), exactly mirrors the direction of association, demonstrated by the corresponding HLA-DRB1 alleles, further corroborating the theory of the HLA-DRB1 locus being responsible for the association. Gluten enteropathy and multiple sclerosis are currently the only exceptions where the primary association with HLA complex is due to HLA-DQ specificities [41].

HLA genes, so far, are not useful diagnostic markers in IgAV [42]. However, the identification of genetic polymorphisms at HLA loci may be useful in determining individuals at higher risk of severe forms of the disease and poor prognosis, which particularly applies to patients with GI involvement and IgAVN, thus providing important information and improving our understanding of pathogenesis and clinical heterogeneity of IgAV.

### Limitations

The main limitation of the present study is the small sample size and, for more accurate results, further studies should be performed on a larger Croatian population of IgAV patients. One potential problem that arises here is the fragmentation of care for patients with IgAV between rheumatologists and nephrologists; therefore, it is important that similar research is based on future collaborations of both specialties. In addition, some of the limitations that can generally accompany HLA studies in IgAV refer to variants in various non-HLA genes associated with immune and inflammatory response, such as genes for cytokines, chemokines, adhesion molecules, T lymphocytes, neoangiogenesis, the renin–angiotensin system and metabolism, that may also contribute to the etiopathogenesis of IgAV and could be associated with different disease phenotypes and disease severity [21,42]. Also, genetic differences within the HLA system are likely influenced by the environment, demographics, racial and regional factors.

## 4. Materials and Methods

### 4.1. Patient Selection and Study Design

A total of 130 children fulfilling the EULAR/PRINTO/PRES classification criteria for IgAV in the period between 2019 and 2022 from three Croatian pediatric rheumatology centers were enrolled in the study [4]. Samples of 3 mL peripheral blood containing ethylenediamintetraacetic acid (EDTA) were taken from each patient with IgAV. Clinical data and laboratory parameters were collected from patient medical records at the time of disease diagnosis until the last follow-up visit. The control group of 202 unrelated healthy individuals from different regions of Croatia was selected to compare the gene and allele frequencies with those obtained from IgAV patients. These subjects were typed for HLA-A, -B, -DRB1 and -DQB1 genes in previous studies [43,44]. Patient(s) and/or their parents or legal guardians of minors signed an informed consent, and study was conducted with the principles of the Declaration of Helsinki.

### 4.2. DNA Isolation

Genomic DNA from whole blood containing EDTA was extracted using a commercial kit (MagNA Pure LC DNA; Roche Diagnostics GmbH, Manheim, Germany) and following the manufacturer’s instructions. All DNA samples were stored at −20 °C.

### 4.3. HLA Class I and Class II Typing

HLA-A, -B and -DQB1 low-resolution typing was performed by the Polymerase Chain Reaction–Sequence-Specific Oligo probes (PCR-SSO) method (Immucor Inc., Stanford, CT, USA) and using the Luminex technology (Luminex Corporation, Austin, TX, USA). The software used for the generation of the HLA data was MatchIt DNA, version 2.5 (Immucor Inc., Stanford, CT, USA). The HLA-DRB1 typing was performed at the high-resolution level using the next-generation sequencing (NGS) method (GenDx, Utrecht, The Netherlands) and using the iSeq 100 Sequencing System (Illumina Inc., San Diego, CA, USA). The obtained results were analyzed using the NGSengine software 2.27.2 (GenDx, Utrecht, The Netherlands).

### 4.4. Statistical Analysis

Continuous data were described as mean and standard deviation (mean ± SD) and categorical variables as percentages. The differences between HLA-A, -B, -DRB1 and -DQB1 gene frequencies, as well as HLA-DRB1 allele frequencies in IgAV patient group, subgroups and controls were examined using the MedCalc software proportions calculator, version 22.014 (MedCalc Software Ltd, Ostend, Belgium) [45], which uses the “N-1” Chi-squared test, as recommended by Campbell [46] and Richardson [47]. The confidence interval is calculated according to the recommended method given by Altman et al. [48]. Additionally, multiple logistic regression analysis was performed to assess the association between the most common HLD-DRB1 genes in several phenotypes of IgAV and the control group. Statistical significance was defined as *p* < 0.05.

## 5. Conclusions

HLA genes are the main genetic factor associated with IgAV pathogenesis, with HLA class II genes being the most strongly associated. Our results also confirm this finding and, additionally, we showed that HLA genes were involved in gastrointestinal manifestations and nephritis, particular disease phenotypes that are highlighted as the most important in IgAV. Therefore, we can conclude that studies, which aim to determine the HLA profile of IgAV patients in a specific population, should be performed in order to define the precise role of HLA alleles but also HLA haplotypes in etiopathogenesis of the disease.

## Figures and Tables

**Table 1:** Demographic and clinical features of patients with IgAV.

Characteristics of the Patients	N = 130	% of the Cohort
**Demographics**		
mean age (years)	6.3 (4.4–8.1)	
female	71	54.6
male	59	45.4
ratio male/female	1:1.2	
**Clinics**		
*Cutaneous manifestations*	130	100
palpable purpura	103	79.2
petechiae	20	15.4
purpura and petechiae	7	5.4
rash extended above waist	57	43.8
bullae, ulcerations, and necrotic lesions	7	5.4
*Joint involvement*	108	83.1
arthritis	32	24.6 *
arthralgias	30	23.1 *
arthritis and arthralgias	46	35.4 *
*Gastrointestinal involvement*	50	38.5
abdominal pain	38	29.2 *
positive FOBT	21	16.2 *
intussusception	3	2.3 *
*Nephritis*	39	30.0
hematuria	12	9.2 *
proteinuria	10	7.7 *
hematuria and proteinuria	14	10.8 *
*Orchitis*	10	16.9 **

Legend: Data are presented as a whole number (%); FOBT—fecal occult blood test; *—expressed as a total number since a patient can have more than one clinical manifestation of affected organ system; **—applicable only for boys.

**Table 2:** The distribution of HLA-A, -B, -DRB1 and -DQB1 gene frequencies in IgAV patient and control groups.

	Gene Frequency (%)		
HLA-A*	**IgAV Patients (N = 70)**	**Controls (N = 202)**	***p* Value (95% CI)**
01	15.0	14.11	/
02	24.3	31.19	/
03	21.4	12.38	0.0092 (2.1–17.1%)
11	7.1	5.94	/
23	1.4	1.73	/
24	10.7	13.12	/
25	2.1	4.21	/
26	5.0	3.22	/
29	0.7	0.99	/
30	1.4	0.74	/
31	1.4	2.48	/
32	3.6	4.21	/
33	1.4	2.72	/
66	0.7	0.25	/
68	2.9	2.48	/
69	0.7	0.25	/
HLA-B*	**IgAV Patients (N = 70)**	**Controls (N = 202)**	***p* Value (** **95% CI)**
07	10.0	9.4	/
08	5.7	9.2	/
13	5.0	3.5	/
14 (B64)	0.0	0.7	/
14 (B65)	0.7	3.5	/
15 (B62)	6.4	4.7	/
15 (B63)	0.7	1.0	/
18	5.0	8.2	/
27	3.6	3.5	/
35	15.0	11.6	/
37	2.9	0.2	0.0054 (0.51–6.9%)
38	4.3	4.5	/
39	1.4	2.7	/
40 (B60)	0.7	2.7	/
40 (B61)	3.6	5.0	/
41	0.7	0.5	/
44	11.4	7.7	/
47	0.7	0.0	/
48	0.0	0.2	/
49	2.1	2.0	/
50	1.4	1.0	/
51	10.7	12.1	/
52	1.4	1.2	/
53	0.0	0.2	/
55	2.9	1.5	/
56	1.4	0.5	/
57	1.4	1.5	/
58	0.7	1.2	/
HLA-DRB1*	**IgAV Patients (N = 130)**	**Controls (N = 202)**	***p* Value (** **95% CI)**
01	13.5	10.4	/
03	7.7	10.6	/
04	9.2	9.9	/
07	8.1	10.9	/
08	4.6	2.5	/
09	0.0	0.2	/
10	1.9	1.0	/
11	15.4	14.9	/
12	3.1	0.7	0.0216 (0.3%–5.3%)
13	12.3	16.3	/
14	7.3	4.5	/
15	9.2	8.4	/
16	7.7	9.7	/
HLA-DQB1*	**IgAV Patients (N = 91)**	**Controls (N = 202)**	***p* Value (** **95% CI)**
02	15.9	19.3	/
03 (DQ7)	20.9	18.1	/
03 (DQ8)	6.6	8.4	/
03 (DQ9)	1.1	2.5	/
04	3.3	1.7	/
05	31.9	25.7	/
06	20.3	24.3	/

Legend: in parentheses—serological equivalent at HLA-B locus; ‘/’—not significant *p* value.

**Table 3:** Distribution of HLA-DRB1 gene (A) and allele (B) frequencies in patient subgroups (IgAV and GI (N = 31), IgAVN (N = 20), IgAVN and GI (N = 19) and IgAV (N = 60)) and controls (N = 202).

Gene/Allele Frequency (%)					
HLA-DRB1*	IgAV and GI (n = 31)	IgAVN (n = 20)	IgAVN and GI (n = 19)	IgAV (n = 60)	Controls (n = 202)
**(A)**					
**10**	6.5	0	2.6	0	1.0
**12**	1.6	2.5	2.6	4.2	0.7
**14**	6.5	17.5	2.6	5.8	4.5
**16**	3.2	15.0	2.6	9.2	9.7
**(B)**					
**04:02**	3.2	0	0	0	2.7
**04:04**	0	2.5	5.3	0	1.2
**10:01**	6.5	0	2.6	0	1.0
**11:01**	9.7	2.5	13.2	4.2	8.4
**11:03**	3.2	0	0	1.7	0.5
**12:01**	1.6	2.5	2.6	3.3	0.7
**14:01P**	6.5	17.5	2.6	5.8	4.5

Legend: IgAV and GI—IgA vasculitis with gastrointestinal tract involvement, IgAVN—IgA vasculitis nephritis, IgAVN and GI—IgA vasculitis nephritis with gastrointestinal tract involvement; IgAV—IgA vasculitis; HLA-DRB1*14:01P—HLA-DRB1*14:01/*14:54.

**Table 4:** The comparison of HLA-DRB1 gene (A) and allele (B) frequencies according to patient subgroups (IgAV and GI (N = 31), IgAVN (N = 20), IgAVN and GI (N = 19) and IgAV (N = 60)) and controls (N = 202).

***p* Value (95% CI)**								
HLA-DRB1*	**IgAV and GI vs. Controls**		**IgAVN vs. Controls**		**IgAVN and GI vs. Controls**		**IgAV vs. Controls**	
**(A)**								
**10**	0.0021 (1.3–14.5%)		/		/		/	
**12**	/		/		/		0.0073 (0.7–8.7%)	
**14**	/		0.0006 (3.9–27.6%)		/		/	
**16**	/		/		/		/	
**(B)**								
**04:02**	/		/		/		/	
**04:04**	/		/		/		/	
**10:01**	0.0021 (1.3–14.5%)		/		/		/	
**11:01**	/		/		/		/	
**11:03**	0.0301 (0.1–10.5%)		/		/		/	
**12:01**	/		/		/		0.0302 (0.1–7.5%)	
**14:01P**	/		0.0006 (3.9–27.6%)		/		/	
	***p* Value (95% CI)**							
HLA-DRB1*	**IgAV and GI** **vs. IgAV**	**IgAVN** **vs. IgAV**		**IgAVN and GI** **vs. IgAV**	**IgAV and GI** **vs. IgAVN**	**IgAV and GI** **vs. IgAVN and GI**		**IgAVN vs.** **IgAVN and GI**
**(A)**								
**10**	0.0050 (1.5–15.5%)	/		/	/	/		/
**12**	/	/		/	/	/		/
**14**	/	0.0242 (1.2–26.4%)		/	/	/		0.0311 (0.9–29.5%)
**16**	/	/		/	0.0311 (0.7–26.1%)	/		/
**(B)**								
**04:02**	0.0485 (0.7–11.0%)	/		/	/	/		/
**04:04**	/	/		0.0117 (0.4%–17.3%)	/	/		/
**10:01**	0.0050 (1.5–15.5%)	/		/	/	/		/
**11:01**	/	/		0.0480 (0.1–23.4%)	/	/		/
**11:03**	/	/		/	/	/		/
**12:01**	/	/		/	/	/		/
**14:01P**	/	0.0242 (1.2–26.4%)		/	/	/		0.0311 (0.9–29.5%)

Legend: ‘/’—not significant *p* value; HLA-DRB1*14:01P—HLA-DRB1*14:01/*14:54.

## Data Availability

All data are available in the manuscript. The datasets generated during and/or analyzed during the current study are available from the corresponding author on reasonable request.
